# Discoveries and advances in plant and animal genomics

**DOI:** 10.1007/s10142-015-0434-3

**Published:** 2015-03-13

**Authors:** Rudi Appels, Johan Nystrom, Hollie Webster, Gabriel Keeble-Gagnere

**Affiliations:** School of Veterinary and Life Sciences, Murdoch University, 90 South Street, Murdoch, Perth, Australia 6150

**Keywords:** Plant and animal genomes, ancient DNA, gene networks, databases, communication

## Abstract

Plant and animal genomics is a broad area of research with respect to the biological issues covered because it continues to deal with the structure and function of genetic material underpinning all organisms. This mini-review utilizes the plenary lectures from the Plant and Animal Genome Conference as a basis for summarizing the trends in the genome-level studies of organisms

## Introduction

Plant and animal genomics is a diverse area with respect to the biological issues covered because it continues to deal with the structure and function of genetic material underpinning all organisms. The Plant and Animal Genome (PAG) Conference held in San Diego (California) in January each year provides an overview across all organisms at the genome level, and often it is evident that investment in the human area provides the leadership, applications and discoveries for researchers studying other organisms. This mini-review utilizes the plenary lectures from the conference as a basis for summarizing the trends in the genome-level studies of organisms, as indicated by the presentations from Phillip Bourne (National Institutes of Health, USA), Beth Shapiro (University of California Santa Cruz), Trey Ideker (University of California, San Diego), Xuemei Chen (University of California, Riverside), Mike Goddard (University of Melbourne, Victoria DEPI), Giles Oldroyd (John Innes Centre, UK) and Christina Warinner (University of Oklahoma). The research covered in this mini-review is based on published papers. Where unpublished information is cited, permission to include the information in this manuscript was obtained from the presenters.

## Evolutionary genomics

Species are the fundamental units in the biological classification of organisms and represent groups of organisms that inter-breed and produce fertile progeny. In the presentation by Beth Shapiro, a detailed genome-level analysis of allele frequencies in some defined populations of polar and brown bears demonstrated that brown bears possess polar bear gene alleles across significant portions of their genomes, whereas brown bear gene alleles appeared to be absent from the polar bear genome (Cahill et al. 2013, 2015). The study was based on sequencing total DNA using either the 100 or 150-bp paired-end chemistry and Illumina HiSeq 2000, assembled using the polar bear genome sequence as a reference (Li et al. 2011). The distribution of allelic variation in both mitochondrial and nuclear DNA was consistent with gene dispersal via male brown bears. The asymmetry in gene allele distribution suggested that hybrid individuals were unable to backcross with the polar bear population. At a broader level, the asymmetric gene allele distribution in admixtures of closely related species has also been found in studies of the hybrid zones between *Mus domesticus* and *Mus musculus* (Teeter et al. 2008). The asymmetry was of a quantitative nature, possibly from asymmetric mating preferences between *M. domesticus* and *M. musculus*, resulting from urine signals recognized between *M. musculus* individuals, but not between *M. domesticus* individuals. The detailed study of Darwin’s finches (*Geospiza fortis*, *Geospiza scandens*, *Geospiza fuliginosa* and *Geospiza magnirostris*) also showed asymmetrical gene flow, which was dynamic and modified by environmental conditions (Grant and Grant 2010).

The asymmetrical gene flows were argued to provide a template for refining the analysis of interactions between climate, ecology and speciation. The plenary lecture by Christina Warinner discussed what is considered to be one of the clearest examples of gene flow influenced by culture/environment in human evolution, namely a genetic adaptation in the regulation of the lactase gene required for the digestion of milk lactose (Burger et al. 2007; Warinner et al. 2014a; Kruttli et al. 2014). The technology used in the studies described by Warinner focused on the ancient DNA and protein from the calculus on teeth of human skeletal remains (Warinner et al. 2014b). The amount of DNA extracted from calculus can be 3 orders of magnitude greater than that obtained from bone or dentine (Adler et al. 2013; Warinner et al. 2014a, b, c), and the tandem mass spectrometric-based analyses for protein has allowed fragments of protein to be interpreted, according to the deduced amino acid sequences of peptides (Warinner et al. 2014a). The technology developments were integral to the expanding field of palaeomicrobiology across a broad range of disciplines. In addition to the analysis of human archaeological faecal and bone/dental samples, the technologies included characterization of oil deposits, and permafrost and deep sea samples (Warinner 2014b). In the case of the human archaeological dental samples, whole genome sequencing of calculus utilized random primers to amplify and sequence a largely unbiased subset of the total DNA. For microbial DNA, the 16S ribosomal RNA (rRNA) amplicons recovered using short primers still provided a valuable source of DNA sequence for classifying the bacteria in the DNA sample under study (Warinner 2014d; current status of genome sequencing in bacteria generally is reviewed in Land et al. 2015, this issue). The analysis of the metagenomic database to assign ancient DNA sequences to bacterial classes and species was still a significant challenge, and it has been noted that the analysis of human gut bacteria using shotgun metagenomic approaches yielded lower diversity estimates than those based on amplicon sequencing of the 16S rRNA gene (Qin et al. 2010). The use of LC-mass spectrometry-based metaproteomics of the protein samples extracted from archaeological specimens, and digested with trypsin, was noted as becoming feasible (Cappellini et al. 2014; van Doorn et al. 2012) because reference datasets for interpreting the peptide sequences were being developed.

The analysis of both dental DNA and protein from human archaeological specimens provided the basis for investigating genetic adaptation in the human population in relation to the regulation of the lactase gene (Kruttli et al. 2014). The lactase enzyme hydrolyzes the lactose in milk to its component monosaccharides glucose and galactose, and persistence of its expression (‘lactase persistence’, LP) in adults is associated with the capacity to consume milk in European, African and Middle Eastern populations without unwanted side effects. The C-to-T single nucleotide polymorphism (SNP), (T-13910) located ca. 14,000 bp upstream of the lactase-phlorizin hydrolase (LCT) gene is associated with an absence of down-regulation of lactase activity after weaning. Assaying the T-13910 SNP in the ancient DNA samples suggested that the increased frequency of this mutation in Europe occurred in the period of 3000 BC to 1200 AD (Kruttli et al. 2014) since it appeared to be missing from the early Neolithic farmers in Europe (Burger et al. 2007). The increased frequency in the T-13910 SNP in the agricultural era in question was consistent with the fact that milk would have provided a clean, versatile and nutritious source of liquid (Lee et al. 1978) in early societies. Independent evidence from the direct assay of the milk whey protein beta-lactoglobulin in archaeological dental calculus in specimens dating back to at least the Bronze Age (ca. 3000 BC) in Europe and northern Southwest Asia (Warinner et al. 2014a) was also consistent with the timing of T-13910 frequency increases. The analysis of dental calculus was thus providing clear indicators for dietary variables driving recent natural selection in humans (Adler et al. 2013; Warriner 2014b).

## Advances in agriculture sciences

The plenary lecture by Xuemei Chen provided new insights into the control of microRNAs with a particular reference to flowering in plants using *Arabidopsis* as a model. It is generally acknowledged that small RNAs (20–24 nucleotides) are involved in the control of gene expression in a wide range of gene networks and that their steady-state levels need to be carefully controlled (Ramachandran and Chen 2008). The family of miR172 microRNAs comprises five genes (MIR172a–e), and the respective promoters bind RNA polymerase II for producing the RNA products that repress the translation of the target *APETALA2* gene transcript (AP2; Chen 2004). The AP2 transcription factor contributes to the gene network that controls the developmental changes from growing stem meristem to forming flowers (Reinhardt and Kuhlemeier 2002; Chen 2004). Reduced levels of AP2 within the gene network are required for this developmental switch. The POWERDRESS (PWR) mutation was found to enhance the expression of MIR172a, MIR172b and MIR172c (leading to reduced AP2 expression) and hence defined one component of the extensive gene network controlling floral determinacy in the stem meristem (Yumul et al. 2013). The expression of MIR172d was independent of PWD. The existence of a family of microRNAs appeared to provide a well-buffered situation to ensure that AP2 protein levels were reduced as required for floral development.

In addition to transcription by RNA polymerase II, the in vivo levels of MIR172 microRNA were also determined by the activity of SMALL RNA DEGRADING NUCLEASE (SDN) genes, in particular SDN3 as judged from the analysis of mutations in the SDN genes (Ramachandran and Chen 2008). The factors controlling the levels of microRNAs more broadly were evidently the result of another network of genes determining the accessibility of microRNAs to 3′-exonuclease activity of SDN protein (Shen and Goodman 2004; Ren et al. 2014). The addition of U residues to the 5′ terminus of microRNA (miRNA) by miRNA nucleotidyl transferase (HESO1 in *Arabidopsis*) triggers their degradation in a process that is closely linked to the binding of miRNA to the argonaute-1 (AGO1) protein, the effector protein implementing the biological properties of miRNA (Ren et al. 2014). Methylation at the 2′-O position of miRNA by the HEN1 protein (in *Arabidopsis*; Yu et al. 2005) protects miRNA from degradation and uridylation (Ren et al. 2014), as judged from the analysis of mutations that suppress HEN1 activity. The wide influence of the regulation of miRNA has been highlighted in the analysis of tetraploid *Arabidopsis* lines (Wang et al. 2006; Ha et al. 2009) where hybrid lines between *Arabidopsis thaliana* and *Arabidopsis arenosa* showed non-additive expression of miRNAs and thus provide one mechanism for the expression of novel combination of genes in polyploids. Non-additive expression of gene per se has been reported in polyploids such as cotton and wheat (reviewed in Appels 2009), and the observations on the networks in play in *Arabidopsis* impact on the analysis of more complex genomes.

The status of genomics in *Arabidopsis* and rice is the most advanced among plants, and in many plant genomes, issues related to long-distance linkage and orientation of genome sequences remain to be a challenge. The new optical mapping technology (BioNano; Cao et al. 2014) has provided significant highlights across a broad range of organisms, and its application to the analysis of a complex genome such as wheat is discussed here as an illustration of the impact of this technology. At the genome sequence level, the International Wheat Genome Sequencing Consortium (IWGSC, http://www.wheatgenome.org/) has tackled the delineation of the genome structure using the capacity to generate bacterial artificial chromosome (BAC) libraries from flow-sorted chromosome arms followed by sequencing of BACs that were arranged into minimum tiling paths (Feuillet et al. 2012). This approach has been complemented by whole genome sequencing of DNA from flow-sorted chromosome arms (IWGSC 2014) as well as DNA from the entire genome (Brenchley et al. 2012; Chapman et al. 2015). A key resource for the assembly of genomes is a detailed reference genetic map since this relates genome sequence data back to the native DNA that exists in a nucleus of an organism (Feuillet et al. 2012). In this context, the optical mapping technologies are particularly significant because they are contributing to bridging the DNA sequence-to-whole chromosome gap (reviewed in Appels et al. 2014). The well-studied human genome has documented numerous structural variations, including 353,126 copy number variations and 1645 inversions, many of which are known to be associated with a wide range of medical issues in patients (Database of Genome Variants, http://dgv.tcag.ca/dgv/app/home). Optical mapping using nano-channels to array molecules of native DNA, plus advanced bioinformatics analyses, has been shown to provide a comprehensive assay system for defining a structural variation in DNA (Cao et al. 2014; O’Bleness et al. 2014). In the analysis by Cao et al. (2014), one labelled Nt.BspQI (nicking endonuclease) site was assayed, on average, every 9 kb in DNA molecules 1 Mb in length. A total of 932,855 molecules larger than 150 kb (223 Gb, ca. 70× average coverage) were studied. In aligning their optical maps to a reference genome sequence, Cao et al. (2014) estimated that the missing label rate was 10 % and the extra labelling rate was 17 %. Comparative analyses showed that the variation in complex regions such as the MHC loci could be readily assayed, in addition to establishing the number of repetitive sequences, INDELS and inversions at biologically significant loci. The optical mapping technology was a key tool in resolving errors in the 1q21.1–q21.2 region of human chromosome 1 (O’Bleness et al. 2014), particularly in the section of the genome carrying the repetitive Neuroblastoma Breakpoint Family (NBPF) genes.

**Fig. 1:**
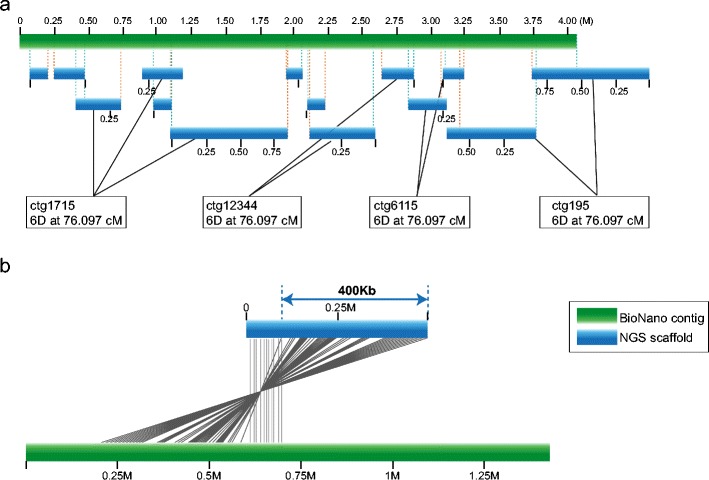
Alignment of sequence scaffolds onto a BioNano contig. Scaffolds derived from MTP BAC clone sequences (Luo et al. 2013) were ordered and orientated onto a BioNano 4-Mb contig spanning (**a**). An example of an error during sequence scaffolding where approximately 400-kb sequence was placed on the opposite side (of correct side) in inverted orientation (*lower panel of figure*), which will ultimately guide editing sequence assembly

The multiple, highly duplicated and complex regions in the human genome that remain largely intractable to analysis with commonly used assembly techniques are also present in other organisms. The application of the BioNano-based optical mapping to the D genome donor of hexaploid wheat (*Aegilops tauschii*) was presented in one of the IWGSC workshops by Mingcheng Luo (UC Davis). The sequence scaffolds (M Luo unpublished, based on Luo et al. 2013) were aligned to the optical maps, and this allowed scaffolds to be ordered and orientated into arrays of several megabases in length (Fig. 1a). Importantly, errors during assembly and scaffolding could be detected and resolved (Fig. 1b).

The genome sequence and annotation in cattle are well advanced, and the plenary lecture by Mike Goddard provided some benchmarks for the analysis of complex traits in cattle, as a model for advances in other organisms including humans and crops. Although the cattle reference genome is still a work in progress, many associations for complex traits such as carcass weight and milk production have been mapped into the high-density SNP maps available for cattle (Saatchi et al. 2014; Goddard 2014) in combination with pedigree studies (Haile-Mariam et al. 2013). The genome-wide association studies (GWASs) carried out on large cattle herds using the 50-K SNP chip (Saatchi et al. 2014) or whole genome sequencing (Daetwyler et al. 2014) has identified many relatively small-effect associations rather than large-effect ones due to rare alleles (Goddard 2014). This has also been found in human GWAS analyses for the complex trait height (Yang et al. 2010, 2011). Increasing the density of SNPs from 50 to 800 K was not found to greatly increase the accuracy of phenotype prediction, but improvements in the analysis using BayesR were found to be useful (Erbe et al. 2012). At a broader level, the comparison of cattle breeds using the 50-K SNP identified regions of extended haplotype homozygosity (EHH) and these regions provided candidate genes for traits that characterize the respective breeds, locating to known QTL for the traits within breeds (Rothammer et al. 2013). The regions of EHH with candidate genes for traits were referred to as selection signatures and could cover up to 12-Mb sections of the genome sequence, but these also included ‘short signatures’ that housed, for example, the MAP2K6 gene that is associated with carcass weight, back fat and marbling in Korean beef cattle (Rothammer et al. 2013). A validation process identified known genes from independent studies, MSTN, within a selection signature region for double muscling in Blanc-Bleu Belge cattle and the gene MC1R within a red coat selection signature region for Red Holstein cattle. Many selection signatures did not provide annotated genes, and this is consistent with the observations that SNP association studies identified small-effect associations with complex traits. However, Rothammer et al. (2013) noted that 97 out of 480 of these signatures with poorly annotated genes overlapped between at least two breeds and could identify regions of particular interest for refining association studies.

The molecular genetic analysis of complex traits in cattle contrasted to the targeted study of nitrogen fixation in crops by Giles Oldroyd in his plenary lecture. The extensive knowledge base in the area of nitrogen fixation in legumes (Oldroy et al. 2011) has provided the basis for new investment in establishing this capacity in crops such as maize, with the view to then transferring to other non-legume crops (https://www.jic.ac.uk/news/2012/07/cereals-self-fertilise/#). It was argued that even the ability to sense nitrogen-fixing soil bacteria and develop simple associations with the roots could be very beneficial to the crop if no other sources of nitrogen were available. The legumes produce specialized organs, nodules, to optimize the symbiotic partnership between plant roots and rhizobia bacteria, but these refined structures were not necessarily a required outcome for nitrogen-fixing bacteria associated with roots to contribute to the supply of nitrogen to cereals.

In legumes, the early stages of nodule formation and bacterial infection require the recognition of the rhizobial signalling molecule, a lipo-chitooligosaccharide, by receptor-like kinases (Oldroyd et al. 2011; Xie et al. 2012). The resulting fluctuation in calcium levels near the nuclei of root hair epidermal cells engages a calcium and calmodulin-dependent protein kinase (CCaMK) that phosphorylates a protein called CYCLOPS. The fluctuation in calcium levels is a characteristic feature of the recognition process and requires the symbiosis receptor-like kinase (SYMRK), components of nuclear pores and two cation channels (SYM pathway; Oldroyd et al. 2011). The next steps in the nodulation process require several transcription factors (NSP1, NSP2, ERN, NIN) to regulate gene expression for nodule formation to begin. The feasibility of establishing these early stages of the nitrogen fixation process in cereals has become evident from the study of arbuscular mycorrhiza (AM) where fungi form branched hyphal structures that penetrate root cortical cell to form a symbiotic relationship that promotes the exchange of nutrients (Brundrett 2002; Kistner and Parniske 2002; Yang et al. 2012). The majority of land plants form AM with zygomycete fungi (order Glomales; Brundrett 2002). Mutation studies in *Lotus japonica* have indicated that at least seven genetic loci control the early stages of establishing both AMs and nodulation (Kistner et al. 2005). Consistent with this finding, Chen et al. (2007, 2008) have shown that both CCaMK and CYCLOPS were required to establish AM in rice and that the rice CCaMK could complement mutations at the DMI3 locus in *Medicago truncatula* to re-establish nodulation. The studies by Gutjahr et al. (2008) showed that the SYM pathway was conserved by selecting rice lines with insertions (mutations) into analogs of the genes coding for CASTOR and POLLUX (required for initiating the Ca-spiking response) as well as insertions into genes coding for CCAMK and CYCLOPS. The analyses of the mutants validated the conclusions that AM colonization of rice roots utilized the same molecular machinery as used to form the early stage of rhizobia nodules.

A point of difference between the early stages of association to form either AM or nodules was the chemical nature of the signalling, namely strigolactone in the case of AM (Bonfante and Requena 2011) and lipo-chitooligosaccharides for rhizobia (Oldroyd et al. 2011; Rival et al. 2012; Chen et al. 2007, 2008). This difference would require a modification of the receptor in cereals in order to recognize the lipo-chitooligosaccharide for attracting the rhizobia and thus utilize some of the components of the molecular machinery for the nitrogen-fixing process that already exist in cereal crops.

## Database analyses

The need to obtain a better value from the large investments in data generation from biological systems was discussed by Phillip Bourne. Laboratory research comprises multiple activities including experimental design, dataset construction, hypothesis formation and hypothesis testing. Formerly, these were usually carried out as integrated activities, but the era of data science is beginning to separate some of these activities (Bourne 2005) and dataset construction can now be carried out in its own right, in an open-ended way. While data publishers often have initial research problems in mind for their data, they might also hope that their data will ultimately be used to study entirely different, unforeseen problems. For example, datasets are now published independently in journals such as *GigaScience* (for example, Craft et al. 2014) and are then referenced in research articles based on the data. This open-endedness addresses the challenge of getting a better value out of large investments in data generation as part of what can be considered to be a digital enterprise (Bourne 2013). A component of this digital enterprise is the improvement and training in accessing methodology in order to work towards a better-shared understanding of how datasets should be produced and consumed. From a technical perspective, ontologies, such as gene ontology (GO, http://geneontology.org/) and EnvO (http://environmentontology.org/), and ontology frameworks, such as Resource Description Framework (RDF, http://www.w3.org/RDF/) and Web Ontology Language (OWL, http://www.w3.org/TR/owl-ref/), go a long way towards enabling the provision of context-independent, widely usable metadata. The adoption of ontology frameworks is a slow but ongoing process in the wider biology research community.

When applying existing datasets to new problems, data users may not always be able to communicate sufficiently with data generators and publishers. In order to maximize the reusability of datasets with limited communication, it is crucial that the datasets are accompanied by well-formulated metadata (Bourne 2005). Good metadata allows future users of a dataset to understand how it was created and how to integrate it with other processes and datasets in a scientifically sound approach. In this way, datasets have maximal mobility and value in their own right, following its separation from the environment in which they were generated.

At the highest and most general level of data integration, the data commons framework provides a conceptual basis for sharing, finding, integrating, reusing and attributing data. Central here is the need to assign a unique identifier (UID) to each dataset, for example a digital object identifier (DOI, http://www.doi.org/). The platform agnostic nature of the data commons framework lets it be deployed in a variety of environments, including a range of cloud platforms (Bourne 2013).

The capacity to obtain a better value from datasets in the area of associating gene networks with particular biological phenotypes was the focus in Trey Ideker’s plenary lecture. Cytoscape (http://www.cytoscape.org/) was discussed as an environment for describing possible gene networks that may exist within the large datasets describing interactions between protein-protein, protein-DNA, kinase-substrate and gene co-expression at the transcriptome level (Saito et al. 2012). The community-based aspect of Cytoscape was facilitated by 152 publically available plug-ins validated by the Cytoscape developers (Saito et al. 2012). The capacity for Cytoscape to utilize gene network information to initiate the analysis of a complex dataset rather than having the gene network as an endpoint was illustrated in the study of several human diseases. The analysis of genotype to phenotype via networks and modules has been reviewed in Carter et al. (2013). Although biological networks described to date, especially in plants, fall short of capturing many important aspects of biological systems, the focus on using available networks and protein-protein interaction (PPI) information more effectively to interpret complex phenotypes has already been proven to be valuable. The network-based stratification (NBS)-based analysis of cancers (Hofree et al. 2013) provided a good example. The extensive database of mutations from the whole-of-genome analysis of tumours determined in The Cancer Genome Atlas (TCGA) and the International Cancer Genome Consortium (ICGC) programs provided the starting point for the analysis. The variation in genome structure such as copy number variation, INDELS in genes and levels of transcription of genes allowed regions of gene networks to be defined that were characteristic of cancer subtypes (Hofree et al. 2013). The projection of genome-scale somatic mutation profiles onto gene interaction networks was built on prior developments in network-based prioritization of disease gene-protein complex associations by Vanunu et al. (2010). The NBS also utilized the consensus clustering software based on resampling (Monti et al. 2003), combining the outputs from 1000 subsamples, to establish clusters of higher confidence. The analysis established a valuable classification diagnostic for cancers.

In the analysis of the complex hereditary phenotype of neurodegenerative motor neuron diseases (hereditary spastic paraplegias (HSPs)) characterized by progressive age-dependent loss of corticospinal motor tract function, Novarino et al. (2014) used whole exome sequencing to identify 18 new candidate HSP genes. The extensive family pedigree analysis of mutations showed that *ERLIN1*, *KIF1C* and *NT5C2* were significant in the HSP phenotype (Novarino et al. 2014). In cases of family-specific mutations, supporting data from zebrafish functional (knock-down) experiments for genes such as *MARS* was obtained. At the network analysis level, the authors developed a HSP interactome network and this indicated that an apparently functionally diverse set of genes were, in fact, closely connected via basic biological processes, involving, for example, the endoplasmic reticulum. The interconnection of networks within the cell has been argued to form modules as part of the hierarchy of distributing function within a cell (Carter et al. 2013; Dutkowski et al. 2013; Mitra et al. 2013; Carvunis and Ideker 2014; Kramer et al. 2014).

## Communication challenges

**Fig. 2:**
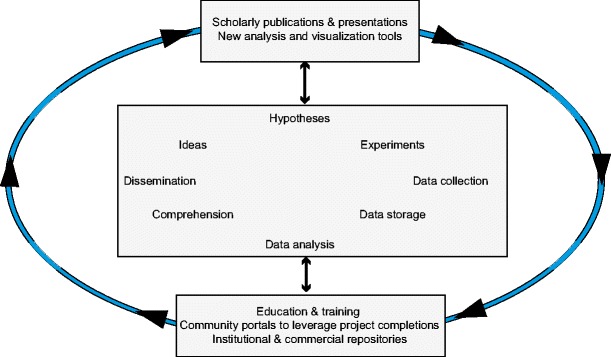
A virtuous cycle based on Bourne (2013) to facilitate defining key aspects of the research cycle for making scientific research activity more sustainable

While essential to research, high-quality datasets are, by themselves, often not sufficient and unlocking the research potential of a particular dataset can sometimes require more sophisticated tools to provide helpful perspectives for lowering the barrier to exploration. As noted above, tools can provide capabilities for visualization, data integration from multiple sources, clustering and prioritization. Phillip Bourne discussed a number of aspects of communication in science in the context of a virtuous cycle which, in part, is shown in Fig. 2 (modified from Bourne 2013) and highlights the key role of data collection, storage and analysis. The sustainability of research investment was argued to rely on the capacity to utilize large datasets in community collaboration, policy and infrastructure development as well as research and training.

The changing role of publications as the primary source of new information was argued to require re-evaluation in light of a prominent role of data collection, data storage and data analysis in the cycle shown in Fig. 2. The investment by funding agencies is usually viewed, by the administrators, as developing datasets and new solutions to issues such as arresting the growth of cancers and releasing crop varieties that are highly yielding when grown in dry environments. The importance of publishing in high impact factor journals was not necessarily rated as significant with the funding agencies as it did with the academics involved in the research.

A good meeting point between the differing views of research funders and research providers is to have the investment in data generation per se being more cost-effective. A key part of this cost-effectiveness was the need to improve the communication that can drive the utilization of large investments in data generation in multiple research projects. An important aspect of this was that high impact journals should take a greater role in ensuring compliance to the uploading of datasets into the digital environments (Alsheikh-Ali et al. 2011; Bourne 2013).
